# An Acute Bout of Self-Myofascial Release Does Not Affect Drop Jump Performance despite an Increase in Ankle Range of Motion

**DOI:** 10.3390/sports8030037

**Published:** 2020-03-19

**Authors:** Mark Godwin, Edward Stanhope, James Bateman, Holly Mills

**Affiliations:** 1 School of Sport and Creative Services, University College Birmingham, Summer Row B3 1JB, UK; j.bateman@ucb.ac.uk (J.B.); h.mills@ucb.ac.uk (H.M.); 2 School of Lifesciences and Education, Staffordshire University, Brindley Building, Leek Road, Stoke on Trent ST4 2DF, UK; edward.stanhope@staffs.ac.uk

**Keywords:** foam rolling, vertical jump, myofascial therapy, massage therapy, stiffness

## Abstract

This study examined the acute effects of self-myofascial release plus dynamic warm up versus dynamic warm up alone on ankle range of motion and drop jump performance. Twenty-five recreationally active participants (male: 16, female: 9) were randomly assigned into a foam rolling (FR) or a dynamic warm up group (CON) (age: 22.8 ± 3.9 years, body mass 75.9 ± 13.2 kg, stretch stature: 174.1 ± 10.1 cm). In a randomised crossover design, each participant completed two experimental sessions that were separated by seven days. Ankle range of movement was assessed while using a weight-bearing lunge test and drop jump performance was recorded via bilateral force plates. Following a 5 min cycle, the foam rolling group undertook self-myofascial release to the lower limb and thoracic/lumbar regions, followed by a dynamic warm up. The control group undertook the same initial warm up plus the dynamic exercises. The level of significance was set at *p* ≤ 0.05. There was a significant increase (*p* < 0.001) in ankle range of motion immediately after the warm up for both groups (pre CON: 37.5 ± 5.31, post CON: 39.8 ± 5.76; pre FR 38.7 ± 7, post FR: 40.3 ± 7.3 deg). No significant difference was found between the conditions (*p* > 0.05). There were no significant differences for any indices of jump performance (*p* > 0.05). Based on these results, foam rolling plus dynamic exercises does not appear to impair or enhance drop jump performance, despite the increases in ankle range of movement.

## 1. Introduction

Increased use of foam rolling and roller massage to assist recovery and to treat post-exercise muscle soreness has been reported amongst a wide range of participants [1]. Furthermore, and noteworthy, is the notion that use of such modalities has advanced beyond the publication of scientific literature [1]. Despite this, foam rolling, a concept of self-myofascial release (SMR), is common amongst therapists and fitness professionals to encourage or promote soft tissue healing [2]. The use of this modality has also been linked to a wide range of outcomes, including the correction of muscular imbalances, reducing muscle soreness, relieving joint stress, improving the efficiency of the neuromuscular system, and increasing range of motion [3]. Halperin et al. reported significant increases in the ankle range of movement following an acute bout (90 s) of self-massage, while using a roller, at both one-and 10-min post intervention with no between group differences (static stretching condition) [4]. However, there was a significant increase in maximal voluntary isometric force at the 10-min point for the roller massage condition only. Su et al., who compared 6 min of foam rolling, static stretching, or dynamic stretching on the sit and reach test in 30 college students, found similar increases in range of movement [2]. The results for each condition showed a significant increase pre versus post; however, the foam rolling condition showed a significantly greater increase between the conditions. Two different protocols that were used by de Souza et al. showed significant increases in ankle and hip range of movement following either two sets of 10 repetitions or two sets of 20 repetitions while using a foam roller amongst 14 recreationally active males [5]. However, another study, a four-week daily intervention of either foam rolling or eccentric exercise, yielded differing results across both groups [6]. At 30 min post intervention, significant increases in ankle dorsiflexion were reported for both groups. Although at four weeks, ankle dorsiflexion only significantly increased for the eccentric exercise group [6]. Despite the differences in self-myofascial release duration across a number of studies, the results highlight its use in acute changes to range of movement.

The use of self-myofascial release yields differing results in relation to performance outcome measures, despite increases in range of movement across a number of studies. Following three sets of 30 s of foam rolling to the gluteals, hamstrings, quadriceps, and calf muscles, a recent study showed no significant change in vertical jump performance at 5, 10, 15, and 20 min post intervention [7]. However, the dynamic stretching and combination group showed a significant increase in vertical jump when compared to both the control and foam rolling group immediately following the intervention. In comparison, no significant differences were found in vertical jump power, vertical jump velocity, knee isometric torque, and hip range of motion following an eight minute bout of lower limb foam rolling to 14 Divisional I football players [8]. However, an earlier study did find significant increases in five out of six outcome measures following an acute bout of foam rolling (five strokes per 30 s across six regions of the upper and lower body) [9]. Similarly, Su et al. showed a significant increase in isokinetic knee extensor performance after 90 s of foam rolling to the lower limbs when compared to a static stretching condition [2]. However, there was also a significant increase in performance for the dynamic stretching group.

Despite the widespread use of SMR, the biological mechanisms behind its effectiveness are limited [10]. More recent work by Young et al. discussed that the plausible benefits of foam rolling may be similar to those of massage i.e., biomechanical, physiological, neurological, and psychological [11]. Furthermore, altered afferent input might be attributed to the activation of mechanoreceptors due to the changes in mechanical tension and pressure whilst foam rolling [11]. Whilst the mechanical effects of foam rolling are not established, Freiwald et al. acknowledges that the mechano- and chemoreceptors are mechanically stressed during foam rolling, which might influence nerve tissue [12]. Others have reported that a muscle will relax following amplified pressure of a foam roller via a decrease in neuromuscular excitability, minimising both myofascial trigger point activity and pain [10]. Of note is the view that the high mechanical loads recorded during foam rolling may be harmful to the connective tissue, nerves, vessels, and bones [12].

The use of plyometric training is well established and evidence suggests that it can be used to increase the vertical jump height for a wide range of participants [13]. Six weeks of drop jump training in addition to regular training resulted in a significant increase in vertical jump height for junior basketball training [14]. Similarly, an eight-week plyometric training intervention, including hurdle jumps and drop jumps, improved both absolute and relative power in soccer players [15]. Whilst men have been reported to show greater improvements to jump height following plyometric training, evidence shows that implementing this type of programme with female athletes also yields greater performances [16]. A 12-week programme, incorporating hurdles, drop jumps, and horizontal jumps, elicited significant increases in jumping ability and kicking speed amongst a cohort of female soccer players [17]. The mechanisms of the stretch-shortening cycle play a role in jump performance and, therefore, contribute to sporting activities [18]. The reactive strength index (RSI) is used to measure the explosiveness of an athlete and a way to quantify the performance of plyometric or stretch shortening activity [19]. In particular, the drop jump can be evaluated while using this index due to the identifiable ground contact time [18]. Subsequent literature has identified the use of a modified RSI (RSImod) to reliably assess this outcome in different athletes [18,20,21]. This method replaces flight with time to take off as the denominator in the standard RSI equation (RSImod = contact time/time to take off) [18].

Therefore, the primary objectives of this study were to determine whether foam rolling to the lower extremities had an effect on vertical jump height drop jump performance and the ankle range of movement. Based on previous literature, it was hypothesised that foam rolling would have an effect on neuromuscular performance and joint range of movement.

## 2. Materials and Methods

A randomised, crossover design was used to test the effect of foam rolling on drop jump performance and range of movement. Each participant undertook a standardised warm up, followed by either a foam rolling intervention or control, to maintain external validity. A number of drop jump related outcome measures were recorded and analysed, including active stiffness, jump height, and modified and standard reactive strength indices. Active range of movement was assessed using the weight-bearing lunge test.

A sample of 30 (male = 21, female = 9) recreationally active participants, all studying a sports undergraduate degree programme, were invited via an announcement on a student virtual learning platform and agreed to participate in this study (age 22.8 ± 3.9 y, body mass 75.9 ± 13.2 kg, stretch stature 174.1 ± 10.1 cm). The participants were eligible for the study if they were physically active and were free from any injury, which might have been exacerbated by drop jump exercise. The participants were excluded if they consumed caffeinated products 12 h prior to the study or participated in lower body fatiguing exercise within 48 h of either testing session. The University Ethics Committee provided institutional ethical approval (Reference: GODW/11/03/19) and all of the procedures were carried out in accordance with the Declaration of Helsinki (Version 2013). Following a verbal and written explanation of the procedures, written informed consent was obtained from all participants prior to any data collection.

### 2.1. Procedures

**Figure 1:**
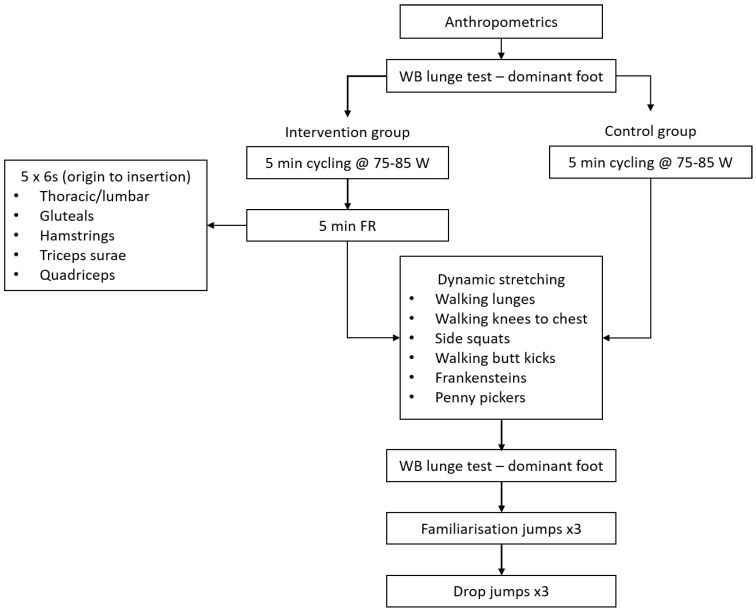
Trial procedures (WB: weight-bearing; FR: foam rolling).

Using a block randomised crossover design (block size of 4), the participants completed two experimental sessions that were separated by seven days (Figure 1). An independent researcher concealed the block size and initially allocated participants to one of two groups, foam rolling (FR) or control (CON). The participants were instructed to refrain from any lower body fatiguing exercise 48 h before the sessions and caffeinated products were prohibited 12 h before any testing session. Water was allowed ad libitum throughout both sessions. The testing briefly consisted of a 5 min warm up followed by either foam rolling plus dynamic warm up or dynamic warm up alone (Figure 1). The active range of movement was assessed using a weight-bearing lunge test and completed prior to and immediately after the warm up using a digital goniometer for the dominant foot. Ecological validity was maintained by combining the intervention with a standardised warm up and not in isolation. The drop jumps took place in a human performance laboratory, with the dynamic warm up and foam rolling taking place in an indoor sports hall.

### 2.2. Measures

#### 2.2.1. Clinometer Validity and Reliability

The validity and reliability of a smartphone digital inclinometer app (Clinometer app, Plain Code, Stephanskirchen, Germany) was assessed using an isokinetic dynamometer (Humac Norm, CSMi Solutions, Stoughton, MA, USA). The dynamometer and app were zeroed in accordance with the manufacturer guidelines prior to any testing. The smartphone (Samsung S9+, Seoul, Korea) was placed on the dynamometer arm throughout the testing and three angles were chosen and selected in a randomised order (15, 40, and 65 degrees). Two researchers, independent of the study, concurrently recorded the angles that were displayed on the smartphone and were blinded to each other. Both researchers were blinded to the angles of the dynamometer. Each angle was assessed on 10 occasions.

#### 2.2.2. Weight-Bearing Lunge Test

The weight-bearing lunge test was used to assess the active ankle dorsiflexion of the dominant foot following a standardised protocol [22]. Starting in a split stance position, the front foot was initially placed 10 cm away from the wall. The participants were instructed to lunge forwards, keeping the knee in line with the second toe, using two fingers of each hand for support on the wall if required. If the heel remained in contact with the ground, the foot was moved further away from the wall in 1 cm increments and the lunge was repeated. Once the heel was unable to remain in contact with the ground, the foot was moved towards the wall in smaller increments until the knee touched the wall. This occurred within 3–4 attempts. The smartphone was then placed at the tibial tuberosity to record the angle of the tibia relative to the ground.

#### 2.2.3. Warm up and Foam Rolling Intervention

The warm up consisted of two or three phases (control or intervention group respectively). Following the weight-bearing lunge test, participants cycled for 5 min on a stationary cycle (Wattbike Pro, Nottingham, UK) (80W, 75–85 RPM). Immediately following the cycle, the participants in the intervention group undertook the foam rolling intervention while using their body weight to provide the pressure. This consisted of five strokes per 30 s for five regions: (1) thoracic/lumbar; (2) gluteals; (3) hamstrings; (4) triceps surae; and, (5) quadriceps/flexors using a hard, hollow core foam roller (GRID^®^ foam roller, Implus LLC, Durham, NC, USA). The foam rolling was performed bilaterally and the entire length of the muscle was covered from origin to insertion [9]. Following the foam rolling or stationary cycle, both groups performed a series of dynamic stretches. A standardised set of dynamic exercises was performed and consisted of six exercises: (1) walking lunges; (2) walking knee to chest; (3) side squats; (4) walking butt kicks; (5) frankensteins; and, (6) penny pickers [23]. Each exercise was performed five times on each leg. Both the intervention and dynamic warm up were completed under the guidance of a qualified therapist.

#### 2.2.4. Drop Jump Protocol

The drop jumps were performed from a 30 cm box onto a set of force plates (Force Decks FD4000, London, UK) with a sampling frequency of 1000 Hz. Data was analysed using the manufacturer’s software (Force Decks Dual Force Platform Hardware and Software Solutions). Three familiarisation repetitions, while using standardised key points, were performed and any errors were addressed using corrective cues prior to the drop jump assessment [24]. For the three drop jumps, participants were instructed to step off the box and upon landing, immediately perform a maximal vertical jump. Hands were kept on the iliac crest and the same lead foot was used when stepping off the box for the three jumps. Two minutes of rest was given between each jump. Lower limb stiffness was automatically calculated by the software and defined as the change in vertical force divided by the displacement of the countermovement during the eccentric phase. Maximal vertical jump height was calculated while using the flight time method. The modified reactive strength index (RSImod) was calculated by flight time divided by the time to take off and the standard reactive strength index (RSI) was calculated by the jump height divided by flight time. The highest score from the three vertical jumps for each condition was used for the analysis.

#### 2.2.5. Statistical Analyses

Pre-designed spreadsheets were used to assess the validity and reliability [25]. Concurrent validity was calculated for the smartphone digital inclinometer app as compared to an isokinetic dynamometer, while using linear regression utilizing Pearson correlation coefficient (*r*) and standard error of the estimate. An intraclass correlation coefficient (ICC) was calculated to measure the intertester reliability for reading the digital inclinometer. Finally, an ICC was calculated to test the reliability of the drop jumps using the three jump scores for the control condition.

Descriptive data were calculated for anthropometric measurements and expressed as mean ± standard deviation. The Shapiro–Wilk’s test was used to assess normality. Lower limb stiffness violated normality and a Wilcoxon signed rank test was used. Paired t-tests were used for vertical jump height, RSImod, and RSI analyses. Mauchly’s test revealed a violation of sphericity for ankle dorsiflexion and Greenhouse–Geisser calculations were subsequently performed. Ankle dorsiflexion was analysed using a two-way mixed model analysis of variance (ANOVA) [condition (foam rolling and dynamic stretching vs. dynamic stretching) × time (pre vs. post)]. All of the data were analysed using JASP (Version 0.11.1, University of Amsterdam, Amsterdam, The Netherlands). The alpha level was 0.05 for all tests and the magnitude of effect for the paired tests was expressed as effect sizes (ES) using Cohen’s *d* and interpreted as small, *d* = 0.2, medium, *d* = 0.5, and large, *d* = 0.8 [26]. The effect size for the ANOVA was reported using eta squared [27].

## 3. Results

Of the 30 participants initially recruited, 25 completed the study (male = 16, female = 9). Three participants voluntarily withdrew from the study, one was withdrawn due to injury, and one consumed caffeine prior to the second trial.

### 3.1. Validity and Reliability

The results showed very high concurrent validity of the smartphone digital inclinometer app when compared to the isokinetic dynamometer (*r* = 0.99, standardised typical error = 0.02, 95% CI = 0.01, 0.02). Intertester reliability for reading the inclinometer was also very high (ICC = 1.0). The reliability of the three control drop jumps was very high (ICC = 0.94, 95% CI = 0.88, 0.97).

### 3.2. Neuromuscular Performance

**Figure 2:**
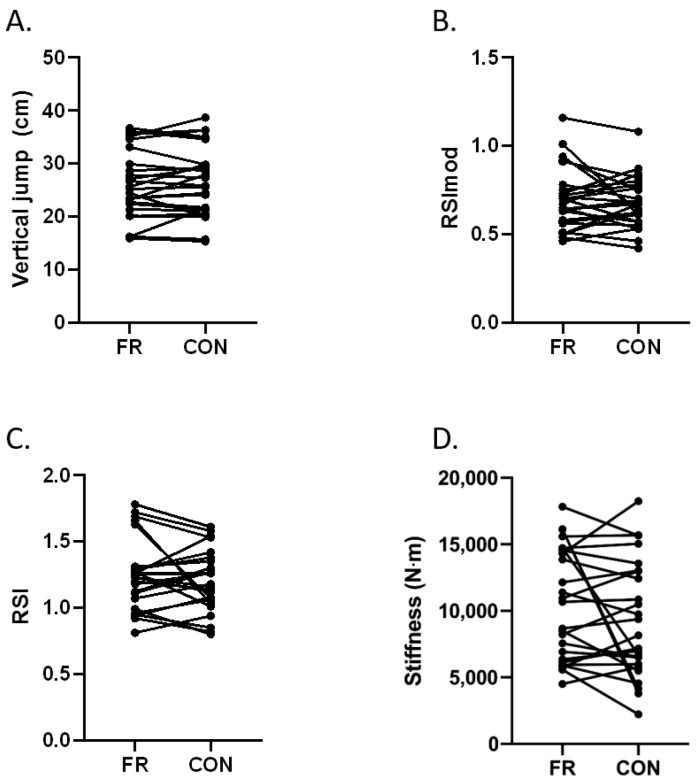
Neuromuscular performance results (FR: foam rolling; CON: control; RSImod: modified reactive strength index; RSI: reactive strength index).

There were no significant differences found for vertical jump (FR 26.06 ± 6.4 vs. CON 26.39 ± 6.5 cm, t(24) = −0.706; *p* = 0.487; *d* = −0.14), RSImod (FR 0.7 ± 0.18 vs. CON 0.67 ± 0.14 t(24) = 0.838; *p* = 0.4; d = 0.168), RSI (FR 1.24 ± 0.3 vs. CON 1.20 ± 0.2, t(24) = 1.024; *p* = 0.316; *d* = 0.2), or lower limb stiffness (FR 10,521.48 ± 4180.1 vs. CON 9254.58 ± 4350.8 N·m^−1^, Z = −0.901; *p* = 0.367; ES = 0.3). Figure 2 presents the individual results.

### 3.3. Range of Movement

**Figure 3:**
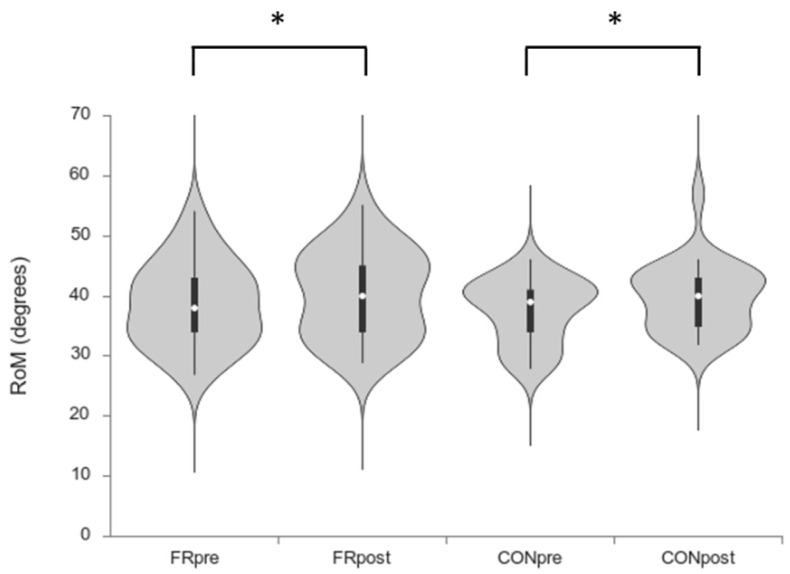
Range of movement results (* denotes significant difference between pre and post only. No difference was found between groups).

The two-way ANOVA revealed a significant difference for time (pre vs. post) for ankle dorsiflexion during the weight-bearing lunge test for both groups (F = 22.9; *p <* 0.001, η^2^ = 0.024). No significant difference was found between conditions (F = 0.22; *p* = 0.64; η^2^ = 0.004) or the interaction time × condition (F = 0.68; *p* = 0.41; η^2^ = 0.001). Tukey post-hoc analysis showed a significant difference between the pre and post foam rolling condition (38.7 ± 7.0 vs. 40.3 ± 7.3º; *p* = 0.04; *d* = 0.4) and the pre and post control condition (37.5 ± 5.31 vs. 39.8 ± 5.76º; *p* = 0.001; *d* = 0.6) (Figure 3).

## 4. Discussion

Rehabilitation and fitness professionals are reportedly using self-myofascial release, utilizing a foam roller, to increase the mobility of myofascial tissue [28]. This study examined whether foam rolling plus a standardised warm up had an effect on drop jump performance and active ankle range of movement. The principle finding of this study showed that a warm up, consisting of 5 min of stationary cycling plus an acute bout of foam rolling followed by a series of dynamic stretching, did not elicit a significant difference in drop jump performance when compared to a standardised warm up protocol in recreationally active participants. However, there was a significant increase in the range of ankle dorsiflexion, pre versus post warm up, when measured using the weight-bearing lunge test for both groups. Both of the measures of reactive strength index showed no difference. Finally, lower limb stiffness was not affected by the foam rolling intervention as compared to the control group.

### 4.1. Range of Movement

A number of studies have demonstrated increases in the range of movement following bouts of differing durations of foam rolling, which is consistent with the results of this study [2,6,7,8,29,30,31]. One study showed no difference between foam rolling and dynamic warm up conditions for sit and reach distance [9]. However, these findings are limited, as there was no analysis of pre versus post measures. Su et al. showed an 11.8% increase in the sit and reach performance following 90 s of foam rolling to the quadriceps and hamstrings [2]. At 2 and 10 min post intervention, MacDonald et al. reported significant increases, 13.7% and 11.3%, respectively, in passive knee flexion following 120 s of foam rolling to the quadriceps [30]. A greater (22%) significant increase in passive ankle dorsiflexion was found following 3 min of foam rolling to the right medial head of the gastrocnemius muscle [31]. Conversely, Škarabot et al. showed a non-significant difference in passive range of motion at the ankle in a FR only group when compared to a static stretching and a combination group [32]. Foam rolling appeared to enhance ROM in the majority of studies, despite differences in the warm up procedures across groups. Our results showed a significant (4%) increase in ankle dorsiflexion following the acute bout of foam rolling plus dynamic warm up. However, the standard warm up group also significantly increased their range of movement (6%). The two studies that showed no difference in ROM were foam rolling alone, with no additional dynamic activities [8,32]. By contrast, Kelly and Beardsley did find a significant increase in the weight-bearing lunge test following three bouts of 30 s foam rolling to the plantar flexors; this increase remained up to 20 min post intervention [29]. Therefore, our study supports the view that foam rolling coupled with a dynamic warm up or dynamic warm up alone increases ROM.

Changes in tolerance and passive properties of muscle may account for ROM alteration [33]. These changes are reported to include muscle tendon unit stiffness and muscle tendon junction displacement [33]. However, Yoshimura et al. reported no changes to fascicle length and aponeurosis displacement following 3 min of foam rolling to the medial head of the gastrocnemius, at a force ranging between 15–25% of body mass [31]. Heat that is generated during the warm might also explain changes in ankle dorsiflexion. In a study applying hot packs for 15 min to the plantar flexor muscles followed by static stretching, the results showed an increase in both passive and active ROM [34]. This increase in temperature accelerates metabolism and circulation and might contribute to an improvement in performance [33]. Whilst muscle temperature was not measured in this study, there is sufficient evidence to support the effects of increased temperature on flexibility that should not be discounted [35]. Bradbury-Squires et al. proposed a possible neural explanation to explain the increase in ROM [36]. They showed that an increase in muscle electromyography activity during roller massage could reach a threshold that is similar to that of proprioceptive neuromuscular facilitation stretching. In turn, this might alter the muscle-spindle length or stretch perception. However, they did note that the amount of pressure that is required to induce an increase in ROM is unknown [36]. Grabow et al. reported no differences in active ROM between differing relative loads (15, 21 and 27% of body mass) [37]. There was however, a significant increase in ROM for all loads immediately and 10 min following the foam rolling (7 and 6.9%, respectively), despite an increase in the rating of perceived pain. Another consideration is the effects of movement on the viscoelasticity and thixotropic properties of muscle. Being commonly used to describe the properties of gel, which becomes fluid-like when shaken or stirred, stiffness or viscosity are therefore dependent on previous movement history [38]. Lakie and Campbell describe how muscles change their mechanical properties following movement, but also return to their original state on cessation [39]. From a myofascial release perspective, an increased extensibility of fascia might be seen as the ground substance becomes less viscous due to the mechanical effects of SMR [40].

### 4.2. Neuromuscular Performance

A number of studies have revealed conflicting results relating to SMR and jump performance [7,23,37,41,42,43,44]. Of note, Sagiroglu et al., reported inhibitory effects in the countermovement jump following an acute bout of SMR at 15 s, 2, 4, 6, 10, 15, and 30 min following the pre-test, despite increases in the seat and reach distance [44]. The SMR protocol used four of the same exercises for the same duration as the current study. However, there was no dynamic stretching and the light aerobic warm up consisted of jogging not cycling. Similarly, Phillips et al. revealed a significant decrease in vertical jump height following 5 min of SMR as compared to pre scores (5.1%) [43]. Notwithstanding this difference, they showed no differences in agility performance. However, in similar results to our study, Grabow et al. showed no difference in drop jump performance following a 60 s bout of roller massage, applied while using a custom made, constant pressure roller apparatus, at a range of relative loads [37]. Likewise, Healey et al. and Mikesky et al. reported no differences in vertical jump after foam rolling was applied to the lower limbs and back [23,42]. Jones et al. also showed no differences in vertical jump performance following 2 min of bilateral foam rolling to the gastrocnemius, quadriceps, hamstrings, and gluteal muscles [41]. A combination group of dynamic stretching and foam rolling, along with a dynamic stretching alone group showed significant increases in countermovement jump immediately and at 5 min post treatment compared to baseline measures [7]. There was no difference in the foam rolling only group. The foam rolling intervention was similar to the current study. However, the duration was greater; three sets of 30 s as compared to a single set. In addition, our study included an additional repetition for the dynamic warm up along with two further exercises.

Foam rolling might also influence muscle stiffness. Calculated as muscle force/muscle length, stiffness is the resistance of a muscle to increase in length and it can relate to a single muscle fibre or the entire body [45]. Morales-Artacho et al. showed significant relative decrements in shear modulus for cycling and mixed groups (cycling and foam rolling) at 5 and 30 min, with the foam rolling group showing a significant decrease at 5 min only [46]. This might highlight the transient effect of foam rolling on stiffness. Our study did not report any differences in active stiffness immediately following the SMR in contradiction to their findings. However, the duration of the foam rolling in the current study was 12.5 min, covering the lower limb and thoracic/lumbar region. By comparison, Morales-Artacho et al. took 15 min SMR on the hamstrings only [46]. Baumgart et al. revealed differences in stiffness at numerous time points and different joints following 2 × 30 repetitions of foam rolling [47]. The stiffness of the thigh was significantly reduced immediately following the intervention, but returned to pre levels at 15 and 30 min By contrast, ankle stiffness significantly increased at 30 min when compared to pre and immediately post intervention [47]. There is a paucity of evidence reporting changes to active muscle stiffness following a bout of SMR. However, some studies have reported the effects of massage on passive stiffness. Eriksson Crommert et al. showed that 7 min of deep massage significantly decreased shear elastic modulus of the gastrocnemius muscle [48]. By contrast, Thomson et al. reported no differences in ankle dorsiflexion passive stiffness across a number of loads following 10 min of petrissage (kneading) massage [49]. Overall, there appears to be conflicting evidence related to the effect of massage on muscle extensibility [50].

### 4.3. Considerations

The proponents of myofascial release highlight that the application of low load, long duration forces manipulate the myofascial complex with the intention to restore optimal length, reduce pain, and improve function [51]. Freiwald et al. discussed the lack of clarity relating to nomenclature, definitions and anatomy of fascial connective tissue, and poor evidence of the myofascial lines [12]. Furthermore, inconsistencies across a range of studies make it difficult to formulate a consensus, despite the claims made by advocates of the procedure [5]. The biomechanical load that was placed on the tissue during the foam rolling and duration might play a role in the effectiveness of SMR. Load was not measured in the current study, but it might contribute to the changes in ROM. Baumgart et al. measured vertical ground reaction forces when foam rolling was applied to the anterior thigh and calves, recording mean forces between 32 and 34% of body mass in a similar protocol to this study [47]. They showed that the countermovement jump performance was significantly reduced 15 and 30 min post intervention, as compared to pre scores. However, there was no difference immediately following the intervention. There is disparity between studies pertaining to the biomechanical load during SMR protocols. For example, studies by Peacock and colleagues did not specify pressure [9,52]. By contrast, Halperin et al. used a 0–10 scale, instructing participants to apply pressure equivalent to 7 out of 10 [4]. The results from these studies varied and, therefore, the biomechanical load may have contributed to these differences. Baumgart et al. reports that the pressure exerted on the underlying tissues may exceed that used in occlusion studies, which can lead to damage of connective tissue, nerves, vessels, and bone [47]. Therefore, consideration of the biomechanical load used in studies is an important factor in future study design. The duration of the intervention might play a role in the effects of SMR, both from a performance and ROM perspective. Some of the studies used shorter protocols (2 min in total), whilst others have used longer durations [30,42,43]. Whilst the mechanisms remain unclear as to how duration may play a role in increasing range of movement, there is some limited evidence to suggest that prolonged SMR might have no extra benefits in enhancing ROM [43]. Future studies may look to quantify an optimal load and duration to increase ROM without a detriment to performance.

The results from this study show that the inclusion of a single bout of foam rolling, alongside a standardised warm up, elicits no greater increase in ankle ROM than warm up alone. The increased ROM that was observed in both groups did not have any significant impact on the stiffness or drop jump performance in recreationally active participants. Therefore, if the intended outcome of the warm up is to increase ankle dorsiflexion, the foam rolling or warm up alone protocols may both be used without a detrimental effect on subsequent drop jump performance.
